# The CMOS Highly Linear Current Amplifier with Current Controlled Gain for Sensor Measurement Applications

**DOI:** 10.3390/s20164653

**Published:** 2020-08-18

**Authors:** Roman Prokop, Roman Sotner, Vilem Kledrowetz

**Affiliations:** 1 Department of Microelectronics, Brno University of Technology (BUT), Technicka 3058/10, 61600 Brno, Czech Republic; kledrowetz@vutbr.cz; 2 Department of Radio Electronics, Brno University of Technology (BUT), Technicka 3082/12, 61600 Brno, Czech Republic; sotner@feec.vutbr.cz

**Keywords:** current amplifier, current controlled gain, accuracy, linearity, CMOS, electronic control, sensor signal processing

## Abstract

This paper introduces a new current-controlled current-amplifier suitable for precise measurement applications. This amplifier was developed with strong emphasis on linearity leading to low total harmonic distortion (THD) of the output signal, and on linearity of the gain control. The presented circuit is characterized by low input and high output impedances. Current consumption is significantly smaller than with conventional quadratic current multipliers and is comparable in order to the maximum processed input current, which is ±200 µA. This circuit is supposed to be used in many sensor applications, as well as a precise current multiplier for general analog current signal processing. The presented amplifier (current multiplier) was designed by an uncommon topology based on linear sub-blocks using MOS transistors working in their linear region. The described circuit was designed and fabricated in a C035 I3T25 0.35-µm ON Semiconductor process because of the demand of the intended application for higher supply voltage. Nevertheless, the topology is suitable also for modern smaller CMOS technologies and lower supply voltages. The performance of the circuit was verified by laboratory measurement with parameters comparable to the Cadence simulation results and presented here.

## 1. Introduction

The adjustable current amplifiers [1,2] have significant application over many electronic circuits and systems. Several interesting solutions have been introduced during the last decades as shown in comparison Table 1. The development of these devices goes in parallel with development of so-called current conveyors (CC) and their adjustable features [2]. The first attempts were evaluated in works [3,4] as parts of CCs (partial two-port transfer available by the device). Both cases implement a ratio of two bias currents for current gain adjustment. This way of adjustment is used very often also in further solutions. More advanced devices were proposed in [5,6,7,8,9,10,11,12,13,14,15,16,17,18,19,20] for example. They offer interesting features discussed in detail in Table 1 Work [8] presents current amplifier feature allowing gain setting by ratio of resistors (no electronic adjustment is shown). However, this concept of current gain setting (unfortunately of the fixed value) is also possible. This can be extended to adjustable control through the replacement of passive element (appropriate replacement is a digital potentiometer or linear operational transconductance amplifier). Many concepts intend digital adjustment of the current gain [5], [14,15,16,17] that is useful for direct cooperation with microprocessors. However, continuous adjustment of the gain is more beneficial for many applications (smooth tuning of filters or automatic amplitude stabilization in oscillators [18]).

Table 1 shows that the majority of solutions is based on bipolar junction transistors, exploiting the benefit of exponential characteristics specially in the accurate current multipliers discussed below. These bipolar topologies meet well the requirements for the exact functionality, good dynamic range and speed (commonly in tens of MHz and more), but at the cost of a large area and current consumption (standardly not mentioned), which is no longer acceptable in the small CMOS technologies [3,4,7,9,10,11,12,16,17]. A large number of previous concepts (CMOS especially) was not tested experimentally, mostly just simple simulation results are shown (without modeling of real effects in many cases) [5,6,7,8,10,11,13,14,15,17]. Very high input impedance (DC resistive component) as well as low output impedance indicate significant drawback of many previous solutions. Special cases are the solutions presented in [7,15]. However, the adjustability of any parameter, but especially the gain, can be often welcomed for electronic tuning of applications in many cases [12,13,16,17]. The output impedance of current output circuits is the most significant issue because of the parasitic behavior occurring in high-impedance nodes of applications (typically in active filters [9]). We can see that situation of state-of-the-art is insufficient in many BJT-based cases [9,11,15,17]. The CMOS-based topologies have significantly better output features, see [21,22]. Work presented by Esparza-Alfaro [21] demonstrates a concept using two CCs and resistor feedback for current gain setting.

The current-mode multipliers are important for our discussion due to similarity of newly proposed concept also using principle of two-quadrant multiplier. Comprehensive comparison [28,29,30,31,32,33,34,35,36,37,38,39,40,41,42,43,44,45,46,47,48,49,50,51,52,53,54,55,56,57,58,59,60,61,62,63,64,65,66,67,68] in Table 2 indicates missing details and incomplete topologies for full implementation in current multiplication or amplification because structures are not designed as fully symmetrical form. They operate with intentional superposition of DC component or principal topology, not including all important parts, in order to obtain behavior required in this work. However, many works [31,33,47,60,67,68] use single or two partial active devices (specified types of current conveyors) in design of current-mode multipliers and fully symmetrical operation (processing of both signal polarities) is easily possible in this form. Usage of OTA-based structures seems to be also very interesting in these nonlinear designs [32,39,44]. It offers building blocks for many nonlinear functions [69]. Logarithmic-domain-based structures are also known in reported concepts [32]. Also, concepts combining conveyors and operational transconductance amplifiers are known [34,64]. There are many possible design approaches exist. However, each of them can be used differently for various purposes and requirements on resulting circuit and application.

Many works from Table 2 suffer from very limited input range of current (below 100 µA), some solutions operate even in nA range that is really insufficient in many practical cases. Despite extremely low power consumption, there are issues with noise and bandwidth limitations. Advantages of the newly proposed solution include quite large input range of linearity ±200 µA (in comparison with other works in Table 2), very low total harmonic distortion (THD) below 0.25%, extremely low input resistance (below 2 Ω), very high output resistance (4 MΩ), and beneficial power consumption. The linearity error is expressed as the ratio error between the maximum and minimum value of the parameter obtained by derivation of the transfer function in the given linearity range and was measured below 6% for the presented circuit. There are solutions having even lower power requirements, unfortunately, there are costs (bandwidth, input range, DC accuracy, etc.) for such benefit in corresponding works. Unfortunately, many detailed parameters of compared solutions are unknown.

As the objective of this work, the precise highly linear current amplifier has been requested for measurement applications handling current signal, conveniently in current sensor processing circuits, especially for the smart sensor ASIC measurement solution. Currently, the main intended usage of the circuit is an automatic gain control current amplifier for precise harmonic reference generator used in Capacitive sensor measurement. The similar solution of the precise sinus reference source like in [70] is assumed to be used and is just under development. While, the referenced circuit is based on the digitally generated voltage reference signal with tunable low-pass filter, the new solution uses the current reference generator, with fixed frequency filter, followed by the current amplifier with gain controlled by the similar principle as is described in the paper [70]. Due to the demand of the supposed capacitive measurement method, this amplifier was developed serving the very low THD as the main parameter because of the assumed derivative signal processing, emphasizing any higher harmonic components of the reference signal.

Furthermore, many modern sensor applications use a high-quality ADC for digitization of the signal where any signal distortion during the analog pre-processing degrades the performance of the ADC.

Generally, this type of circuit is especially suitable to realize tunable multi-output current-mode generators as well, where good accuracy and low THD is also requested. Amplitude modulation of the current signal is another tailor-made application for the presented circuit. However, the designed amplifier prototype has been optimized for use in precise measurement applications processing high current signals.

With respect to the intended application requirements an objective of the work was to develop and design an adjustable accurate current amplifier for current sensor signal processing, and other precise current measurement application, having fulfilled these features simultaneously:

(a) low total harmonic distortion of the processed current signal as the most considered parameter (<1%)
(b) wide and linear input range (about ±200 µA)
(c) very low input resistance (<10 Ω)
(d) high output impedance (>1 MΩ)
(e) adjustability of current gain (theoretically 0–2)
(f) high linearity of the gain control for current gain B < 0.8
(g) acceptable power consumption (comparing to the process signal current) below 5 mW

## 2. Circuit Principle and Design

**Figure 1:**
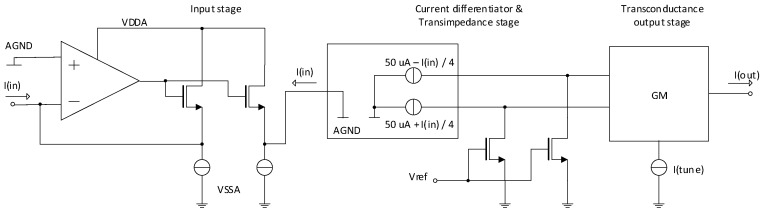
A principal topology of the current amplifier with tunable gain.

Considering all requirements, the idea of the classical quadratic current multiplier topology was rejected mainly because of the high linearity (low THD) demand, simultaneously with wide input range and lower current consumption. With an experience with design of linear or trans-linear circuits using modular approach for compiling the convenient blocks we decided to build-up the current amplifier from a highly linear transimpedance stage, followed by the tunable transconductance stage, both exploiting MOS transistors in their linear region. To meet the requirement of low input impedance a feedback topology based on the differential Miller-OTA amplifier was used. Unfortunately, this solution reduces the frequency bandwidth of the final circuit, but sufficient for many sensor measurement applications. A principal topology of the presented circuit is shown in Figure 1.

The operational amplifier structure at the input provides low impedance for input current I(in). The input current is then copied to the output of the block. In fact, this input block works as a current conveyor CCII. The conveyed input current is converted to two differential currents in differentiator and lowered by factor 4 because of lowering current consumption and transistor area. All the procedure is made by the high accuracy and high output impedance cascoded current mirrors. Differential currents flow to the fixed impedances created by NMOS transistors. As obvious, playing with V_ref_ brings the other degree of freedom to set gain. As it can come with degradation of linearity for unadvised large changes and is not necessary, it stays fixed in the presented design. Voltage signals at the outputs of the transimpedance stage are then processed by the adjustable gm stage. All parts have been designed with maximum respect to linearity and accuracy. Although, the transconductance output stage gives its best THD parameter in the differential connection, therefore the differentiator and differential transimpedance stage was used.

### 2.1. Input Stage and Transimpedance Stage in Detail

The target of this block is to provide low impedance current input and then transfer the input current to defined impedance where it is converted to the voltage driving the following transconductance stage.

The main requirement for the transimpedance stage is to make this conversion as much linear as possible but conveniently also dual to the transconductance stage in the sense of the total amplifier gain stabilization with respect to manufacture and temperature corners. Considering utilization of the NMOS transistor as the active component in the transconductance stage, it has been used also here. As long as the main components of both stages have the same technology parameters (mainly $K_{PN}={µ}_{N}\;.\;C_{OX}$) and temperature characteristics, the effect of corners is minimized.

The full principle turns around the generally known equation for MOS transistor working in the linear region, mentioned in (1),

(1) $$I_{D}=\frac{K_{PN}.W}{L}\;\left[\left(V_{GS}-V_{TH}\right).V_{DS}-\frac{V_{DS}{}^{2}}{2}\right],$$

from which, by solving the quadratic equation, we obtain,

(2) $$V_{DS}=\left(V_{GS}-V_{TH}\right)-\;\frac{\sqrt{{\left(\frac{K_{PN}W}{L}\right)}^{2}.\;\;{\left(V_{GS}-V_{TH}\right)}^{2}\;-\;2\;\frac{K_{PN}W}{L}\;.\;\;I_{D}}}{\frac{K_{PN}W}{L}}\;.$$

Derivation according to *I*_D_ gives the *R*_DS_ value of the MOS serving as the resistor,

(3) $$\frac{dV_{DS}}{dI_{D}}=R_{DS}=\frac{1}{\sqrt{{\left(\frac{K_{PN}W}{L}\right)}^{2}.\;\;{\left(V_{GS}-V_{TH}\right)}^{2}-\;2\;\frac{K_{PN}W}{L}\;.\;\;I_{D}}}\;.$$

From Equation (3) there it is obvious that the *R*_DS_ is dependent on the *I*_D_. For good linearity it is necessary to keep $2.\;\frac{K_{PN}W}{L}.\;I_{D}$ much smaller than ${\left[\frac{K_{PN}W}{L}\;.\;\left(V_{GS}-V_{TH}\right)\right]}^{2}$. Satisfying that condition requires high *V*_GS_ and low *I*_D_ and brings linear MOS transistor resistance of the “ideal” value:

(4) $$R_{DS}=\frac{1}{\frac{K_{PN}W}{L}\;.\;\;\left(V_{GS}-V_{TH}\right)}\;.$$

**Figure 2:**
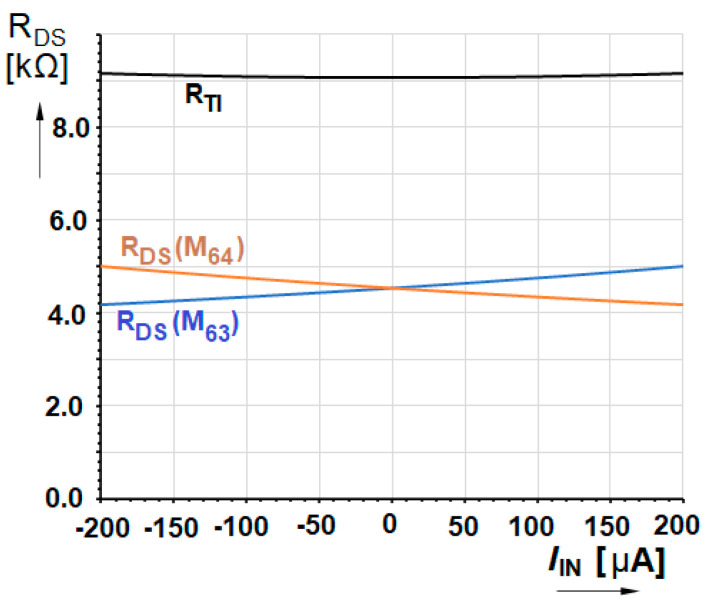
Differential transimpedance as the sum of the linear transistors *R*_DS_ with their dependences on *I*_IN_ calculated by Equation (5).

Unfortunately, when reasonably high current is processed by this way then it is very difficult to keep linearity under 10%. On the other hand, if the differential topology is used at the cell output, the differential output voltage *V*_PN_ = *V*_ZP_ − *V*_ZN_ corresponds to the sum of both linear MOS resistances. However, when *I*_D_ current increases through one of them, as a result of the input current change, the second one decreases simultaneously. It suppresses the parasitic non-linear phenomenon significantly. With respect to the developed presented topology the *I*_D_ current in (3), flowing through the linear transistors, is represented by some part of input current added to and/or subtracted from a pre-biasing current respectively in the individual branches. Considering that the input current *I*_IN_ is reduced 4 times (lower consumption as well as the non-linearity effect) the differential transimpedance exhibits value,

(5) $$R_{TI}=\frac{1}{\sqrt{{\left(\frac{K_{PN}W}{L}\right)}^{2}.\;\;{\left(V_{GS}-V_{TH}\right)}^{2}-\;2\;\frac{K_{PN}W}{L}\;.\;\;(I_{BIAS}+\;\frac{I_{IN}}{4})}}+\frac{1}{\sqrt{{\left(\frac{K_{PN}W}{L}\right)}^{2}.\;\;{\left(V_{GS}-V_{TH}\right)}^{2}-\;2\;\frac{K_{PN}W}{L}\;.\;\;(I_{BIAS}-\;\frac{I_{IN}}{4})}},$$

with opposite effect of the parasitic part for each linear MOS resistance. The graphic solution of this effect is presented in Figure 2.

Now there is seen that the non-linearity reduction depends on the ratio of the fixed part of the equation ${\left[\frac{K_{PN}W}{L}\;.\;\left(V_{GS}-V_{TH}\right)\right]}^{2}-2.\;\frac{K_{PN}W}{L}.\;I_{BIAS}$ and the input current dependent part $2.\;\frac{K_{PN}W}{L}.\;\frac{I_{IN}}{4}$. The *I*_BIAS_ current should be designed as small as possible to maximize the fixed part of the transimpedance. In the presented circuit the *I*_BIAS_ = 50 µA and then the appropriate currents through each transistor vary in range (0 ÷ 100) µA.

**Figure 3:**
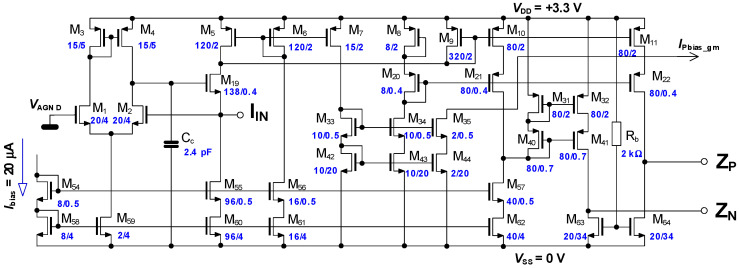
Slightly simplified schematic of the input and transimpedance stage.

The final schematic of the presented input transimpedance stage is shown in Figure 3. The low input I_IN_ impedance is satisfied by operational amplifier consisting of M_1_–M_4_, M_59_ with follower M_19_, working in the unity feedback. M_9_ senses the input current change (as the difference of fixed current of bias source M_60_, while M_5_ makes some small pre-bias due to linearity) and mirrors it by factor 1/4 to current sources M_10_, M_11_. While M_11_ sources the current directly to transimpedance transistor M_64_, the second transimpedance transistor M_63_ is sourced by the complementary current. The output differential voltage *V*_PN_ is taken between the terminals Z_P_ and Z_N_. The realized circuit differs only in matters of ESD protection.

As the sensed input current has been decreased by factor 4 before it is applied to the transimpedance transistors M_63_, M_64_, because of the linearity demand discussed above, then the same factor must be included in the full circuit transimpedance gain equation. The differential output voltage of the whole input transimpedance block is then ideally:

(6) $$V_{PN}=\frac{1}{4}R_{TI}\;.\;I_{IN}\tilde{=}\;\frac{1}{4}\;\;\left(\frac{2}{\sqrt{{\left(\frac{K_{PN}W}{L}\right)}^{2}.\;\;{\left(V_{GS}-V_{TH}\right)}^{2}-\;2\;\frac{K_{PN}W}{L}\;.\;\;I_{BIAS}}}\right).I_{IN}\;\;.$$

The differential transimpedance gain of the realized circuit is calculated as R_TIG_ = 2400 Ω.

**Figure 4:**
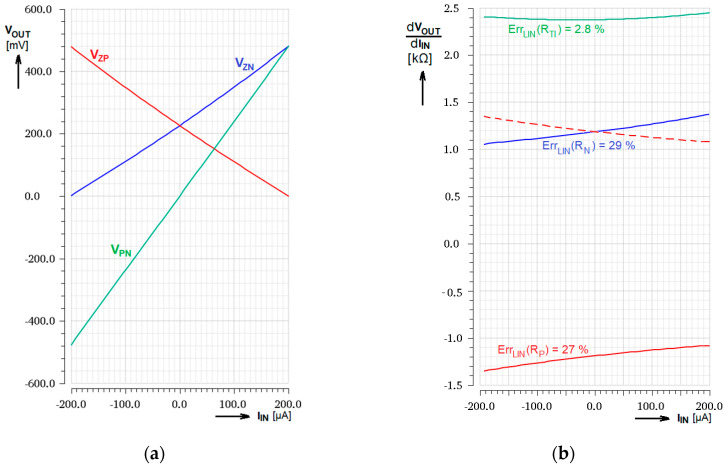
Simulated transimpedance response of the input stage (**a**) Output voltages as the function of *I*_IN_; (**b**) Transimpedance gain as the derivation of the transimpedance transfer functions.

Figure 4a presents the simulated output voltages *V*_ZP_ and *V*_ZN_ together with differential output voltage *V*_PN_ as the functions of the input current *I*_IN_. Derivations of these voltages according to input current, representing the transconductance gain of the whole block, are presented in Figure 4b. The red dashed line represents the transconductance gain of the Z_P_ output branch re-calculated to the differential transconductance gain. Then, it corresponds to Figure 2, considering the factor 4. Due to the differential topology the linearity error is decreased from approx. 28% at one transistor to 2.8% in differential signal.

### 2.2. Tunable Transconductance Stage

**Figure 5:**
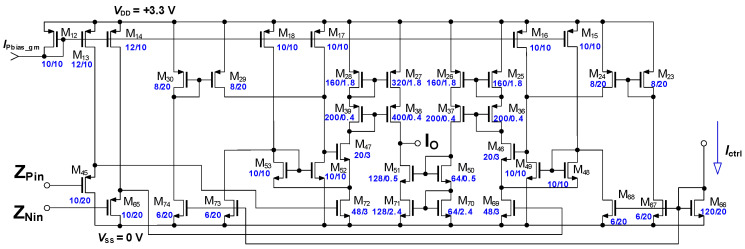
Slightly simplified schematic of the tunable transconductance stage.

As the base topology for the transconductance stage the circuit presented and thoroughly discussed in [71] has been used in its differential connection. This introduced topology was improved, compared to the original version, in tunability, current output impedance and in linearity of the regulation as well. All these improvements have led to the circuit introduced in Figure 5.

The circuit operation consists of a differential connection of two transconducting transistors M_72_, M_69_ working in their linear region. It means, they both work according to the Equation (1) as well. From that equation can be seen that if *V*_DS_ is kept constant then the drain current *I*_D_ is controlled just by *V*_GS_ of the transistor with strongly linear dependence, called transconductance *gm*. The transconductance is then adjusted by *V*_DS_ as a parameter.

Drain to source voltage *V*_DS_ across these transistors must be kept low to stay in triode operation and is equal to the difference between the overdrive voltages of transistors M_53_, M_52_ (and M_49_, M_48_ respectively), which both work in their saturation region and draw a constant current. The feedback loop consisting of transistors M_53_, M_52_ and M_47_ (and M_49_, M_48_ and M_46_) keeps the voltage *V*_DS_ of the main M_72_, M_69_ transistors constant when an input signal is applied [71]. Despite this topology there stays a parasitic effect of the small *V*_DS_ variation (contemplating the branch of M_72_ for instance) caused by *V*_GS_ shift of M_47_ due to the *I*_D_ modulation. It corresponds to the M_47_ transconductance and is compensated by the feedback loop gain. In case of infinity feedback gain the error is zero. Inspecting the real ∆*V*_DS_ of M_72_, we get,

(7) $$∆V_{DS}\left(M_{72}\right)\;\;=\;\;\frac{∆I_{D}\left(M_{72}\right)}{gm(M_{47)}\;.\;\;A_{FBloop}}\;\;=\;\;\frac{\frac{K_{PN}.W.\;V_{DS}}{L}\left(M_{72}\right)\;.\;\;∆V_{GS}\left(M_{72}\right)}{\sqrt{\frac{2I_{D}K_{PN}W}{L}}\left(M_{47}\right)\;.\;\;\sqrt{\frac{2I_{D}K_{PN}W}{L}}\left(M_{52}\right)\;.\;\;r_{d}}\;\;\;,$$

where *r*_d_ is a dynamic impedance of the net of M_52_ drain and together with transconductance of that transistor they define the feedback gain. As it should be kept high the lower currents and long channels of the connected transistors are recommended.

In the presented case of the differential topology usage the equation for transconductance stage output current is then as follows, assuming *V*_INdiff_ is the differential input voltage of the stage,

(8) $$I_{OUT}={\left(\frac{K_{PN}.W.\;V_{DS}}{L}\right)}_{\left(M_{72}\right)}.\;V_{INdiff}+\frac{{\left[{\left(\frac{K_{PN}.W}{L}\right)}^{2}.\;\;V_{DS\;.\;\;}{\frac{V_{INdiff}}{2}}^{2}\right]}_{\left(M_{72}\right)}}{\;gm(M_{47)}\;.\;\;A_{FBloop}\;}+\frac{{\left[{\left(\frac{K_{PN}.W}{L}\right)}^{2}.\;\;V_{DS}{}^{2}\;.\;\;\;\frac{V_{INdiff}}{2}\;\;\right]}_{\left(M_{72}\right)}}{\;gm(M_{47)}\;.\;\;A_{FBloop}\;}$$

        
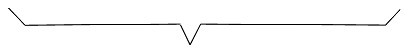
       *Linearity error*

with $A_{FBloop}=gm\left(M_{52}\right).\;r_{d}$ (and complementary about M_49_ in the opposite branch too) as the gain of the closed feedback loop that regulates voltage across the main linear transistors as it is set by difference of the M_53_ and M_52_ drain-to-source voltages. The respective *r*_d_ is dynamic impedance of the nets where drains of M_52_ (M_49_) are connected to and can be calculated as the parallel connection of all impedances connected to that net. For the discussed case it can be expressed as the appropriate MOS transistors output impedance combination like $r_{d}\left(M52\right)=\frac{r_{DS}\left(M52\right)\;.\;r_{DS}\left(M17\right)\;.\;\;r_{DS}\left(M29\right)}{r_{DS}\left(M17\right)\;.\;\;r_{DS}\left(M29\right)+r_{DS}\left(M52\right)\;.\;\;r_{DS}\left(M29\right)+r_{DS}\left(M52\right)\;.\;r_{DS}\left(M17\right)}$.

The deliberation of the Equation (8) gives us the recommendations for design to minimize the linearity error. For the best linearity result we need to keep *V*_GS_ of the M_72_, M_69_ high and their *V*_DS_ sufficiently low to keep these transistors in true linear region. Simultaneously the high *gm* of M_47_, M_46_ is convenient as well as the feedback gain *A*_FBloop_.

In [71], just modifying the current through M_52_ (M_49_) causes the *V*_DS_ of M_72_, (M_69_) change and in this manner it allows to tune transconductance parameter *gm* of the circuit. Unfortunately, the higher tunability needs a high range of the control current within which tuning nonlinearity comes. The presented improved transconductance circuit changes both appropriate currents differentially, through current sources consisting of M_73_, M_74_, M_30_, M_29_ (and M_67_, M_68_, M_23_, M_24_ in the opposite branch) which increase/decrease the current in both transistors, setting the ∆*V*_DS_ across the main linear MOS. It brings high *gm* tuning range as well as linearity of the control with lower control current *I*_CTRL_ amplitude. Another improvement was done in the output stage to increase current output impedance by cascoded mirror as well as input level-shifter adapting circuit to the more convenient input range and setting the higher quiescent *V*_GS_ of the M_72_, M_69_ to ensure the linear region.

## 3. Results

The introduced amplifier has been designed and thereafter fabricated by the Europractice project in ON Semiconductor I3T25 process suitable for the intended application. Features of this circuit were verified by experimental measurements and compared with the design simulations. The obtained parameters, and simulation to measurement comparison, are given in this chapter.

### 3.1. DC Transfer Characteristics

**Figure 6:**
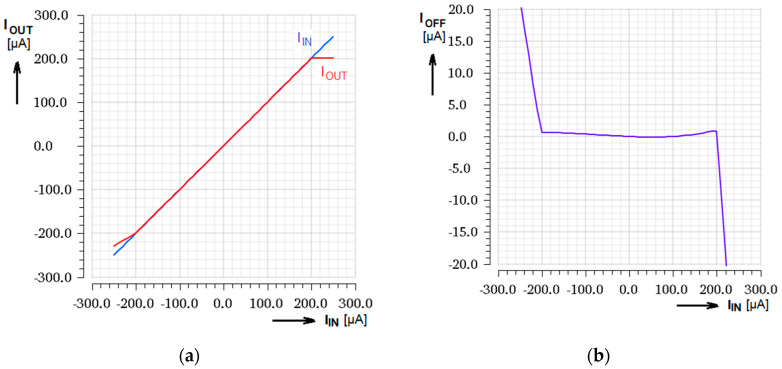
Simulated DC transfer response; (**a**) Input and output currents for amplifier gain B = 1; (**b**) Current input offset on input current dependence.

As the basic test of functionality, input range and systematic input offset, the input to output transfer function characteristic together with the input current asymmetry graph can be used conveniently. Figure 6 shows the input and output currents simulated together in one graph for amplifier gain set to 1 and systematic input offset for typical process. There is the limitation of the input current at ±200 µA, due to the differentiator topology, clearly seen. For another process corner the value can be slightly different. The systematic current input asymmetry, when *I*_IN_ = 0, is typically 5.4 nA.

**Figure 7:**
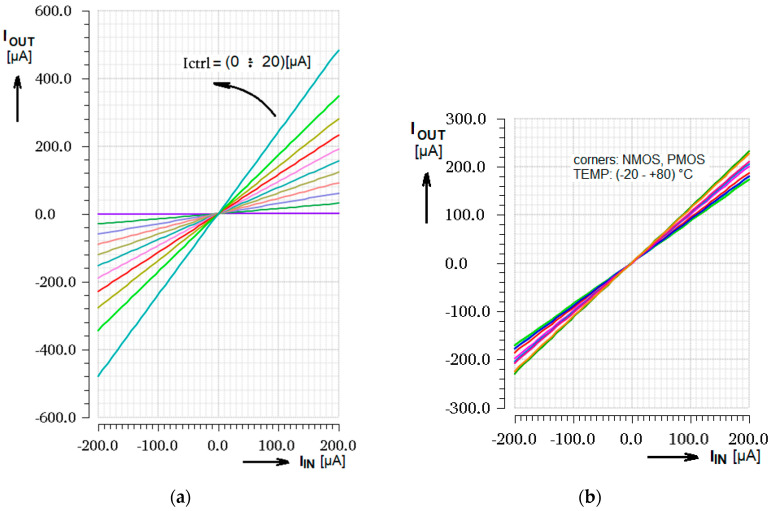
DC transfer response; (**a**) Parametrical simulation for input current range ±200 µA and stepped gain setting current *I*_CTRL_ from 0 to 20 µA; (**b**) Corner analysis for typ. current gain B = 1 (*I*_CTRL_ = 12.5 µA).

The same response simulated while the gain control current *I*_CTRL_ is stepped from 0 to 20 µA with step 2 µA, presented in Figure 7a, gives a hint by curves slope that the control of the gain is highly linear up to *I*_CTRL_ = 12 µA which represents a gain close to 1. Corner analysis across the process and temperature range of (−20 ÷ +80)°C, when current gain is typically set to *B* = 1, is shown in Figure 7b. The tolerable gain dispersion in range ± 15% is achieved by using the same device type (NMOS) for transimpedance and conductance operation simultaneously.

**Figure 8:**
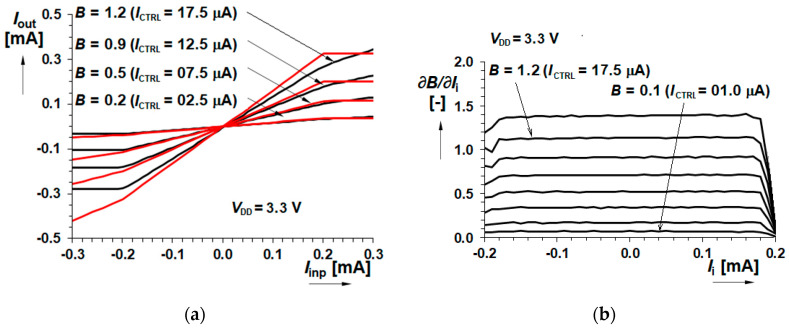
Measured DC transfer response (**a**) Comparison of the simulated (red) and measured (black) curves; (**b**) Measured gain across the input current range.

The comparison of the simulated and measured results offers Figure 8. The graph on the left compares DC transfer results of the real tested device with the theoretical characteristics. The right-side graph introduces the gain value at different input currents. This provides information with respect to the input signal linearity if the gain is constant across the input current range and defines the linear input range. From the comparison there is seen that the investigated real device has a slightly smaller gain and input current range against the simulation, but both parameters are in the scope of corners and presumably correspond to the smaller internal bias current.

**Figure 9:**
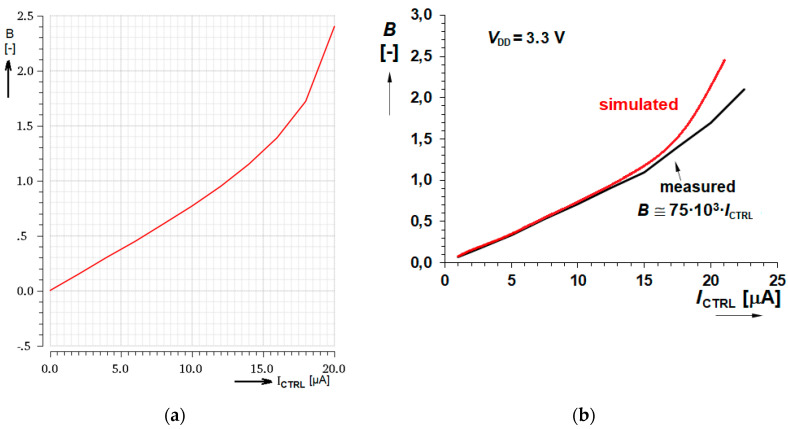
Gain control characteristics (**a**) Typical Cadence simulation of the gain controlled by current *I*_CTRL_; (**b**) Simulation and measurement comparison.

The graph of the gain control by *I*_CTRL_ is displayed in Figure 9 where both simulated as well as measured curves are given. Let us notice the excellent linearity up to *I*_CTRL_ = 10 µA and good linearity to *I*_CTRL_ = 12.5 µA.

### 3.2. Input Signal Linearity and THD

**Figure 10:**
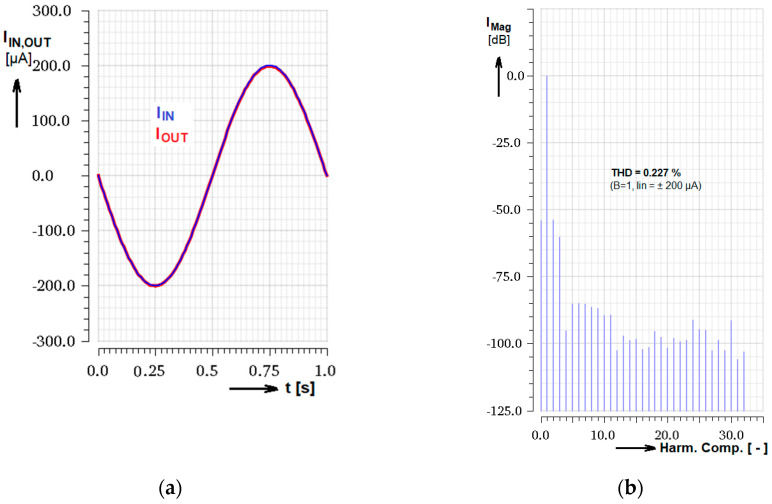
DFT analysis of the output harmonic current; (**a**) Input and output signal transient simulation for the gain set to B = 1; (**b**) DFT analysis expressed in dB related to the first harmonic component and appropriate THD result.

Linearity of the current transfer was the most important parameter considered during design of the amplifier and many other properties, namely frequency bandwidth, were sacrificed to that. The simulated input and output current sinus signal transient as well as the DFT analysis can be seen in Figure 10 for the case of input signal amplitude *I*_IN_ = ± 200 µA and current gain B = 1. As the result the total harmonic distortion THD = 0.227% was calculated by this simulation. Linearity of the signal transfer taken from the simulated DC transfer characteristics for *I*_IN_ = ± 200 µA (corresponding to Figure 6) and *I*_CTRL_ from 10 µA to 20 µA was evaluated in the range of (1.17 ÷ 1.8)%. (As an informative result it can be mentioned that the Cadence simulation values of the transconductor stage itself, as the main tunable part, show the THD_gm_ = approx. 16 m% for the small input signal [mV] and THD_gm_ = approx. 0.25% for large input signals).

**Figure 11:**
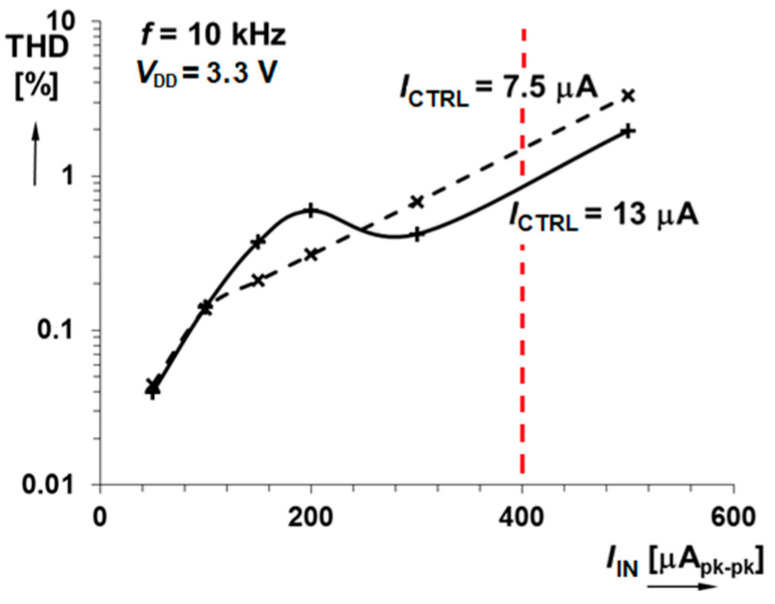
Measurement of output signal THD dependence on the input signal amplitude for two gain control currents *I*_CTRL_. Let us note that the maximum designed input peak-to-peak range is *I*_INmax[PK-PK]_ = 400 µA.

In addition to the simulation, DFT measurement was made to express the parameter in relation to the input signal amplitude for two *I*_CTRL_ currents (see Figure 11). Let us note that the maximum designed and relevant peak-to-peak input range is *I*_INmax[PK-PK]_ = 400 µA.

### 3.3. Linearity of the Control and Control Input THD

Although, strict linearity is often not necessary for the controlled amplifiers, especially in the case of automatic feedback regulation, in some sensor applications it can be beneficial, for example in amplitude modulation processing or the current multipliers. Exploiting the results from Figure 9 we could define the gain control linearity up to 10% for *I*_CTRLmax_ = 12.5 µA (B = 1), as the maximum *I*_CTRL_ value considered during design.

**Figure 12:**
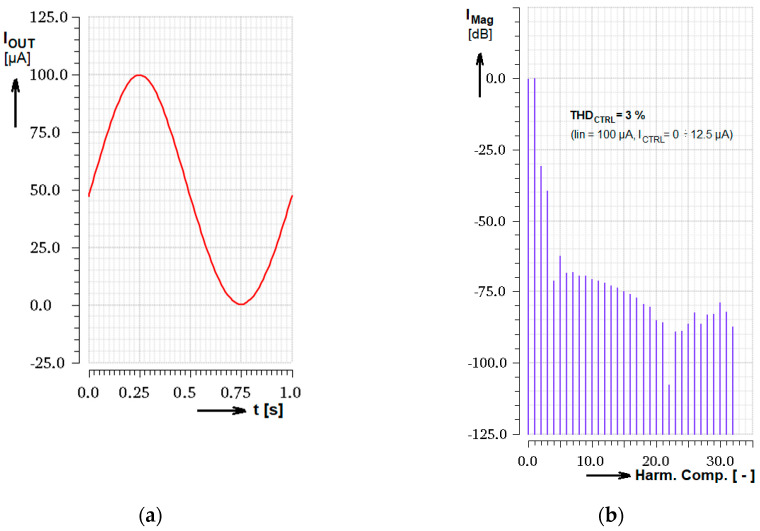
Simulated DFT analysis of the signal transferred from the gain control input; (**a**) Output current transient for *I*_IN_ = 100 µA and the gain harmonically modified from 0 to 1; (**b**) DFT analysis expressed in dB related to the first harmonic component.

The experimental use of the control current input as the signal input was simulated and obtained results are presented in Figure 12. In this test the main input current was set to constant *I*_IN_ = 100 µA and control current *I*_CTRL_ was driven by the harmonic signal from 0 to 12.5 µA (with the DC pre-bias of 6.25 µA). From the transient output response, the DC shift of the output signal can be seen, as well as from the DFT results. Cadence evaluated value of THD = 3%.

### 3.4. Frequency Response and Bandwidth

**Figure 13:**
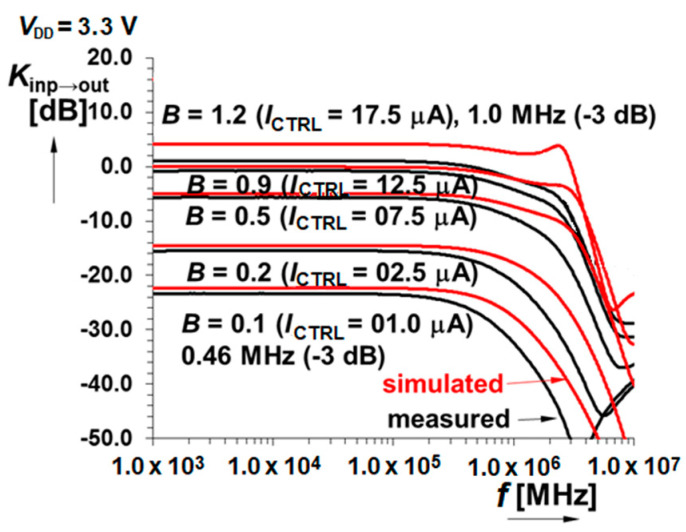
Frequency transfer characteristics with measured bandwidth in the scope (0.46 ÷ 1.0) MHz.

As was already mentioned earlier, the frequency bandwidth was sacrificed to other parameters of the amplifier and the high bandwidth is often not the main request of sensor applications. Despite that, the frequency response of the gain is presented (see Figure 13) comparing simulated and measured results. The values of the frequency bandwidth for different gain will be given in the results overview Table 3.

### 3.5. Input Current Offset

Due to the input offset is one of the important aspects for the circuits used in the measurement applications, dealing with this item must be part of the analysis.

**Figure 14:**
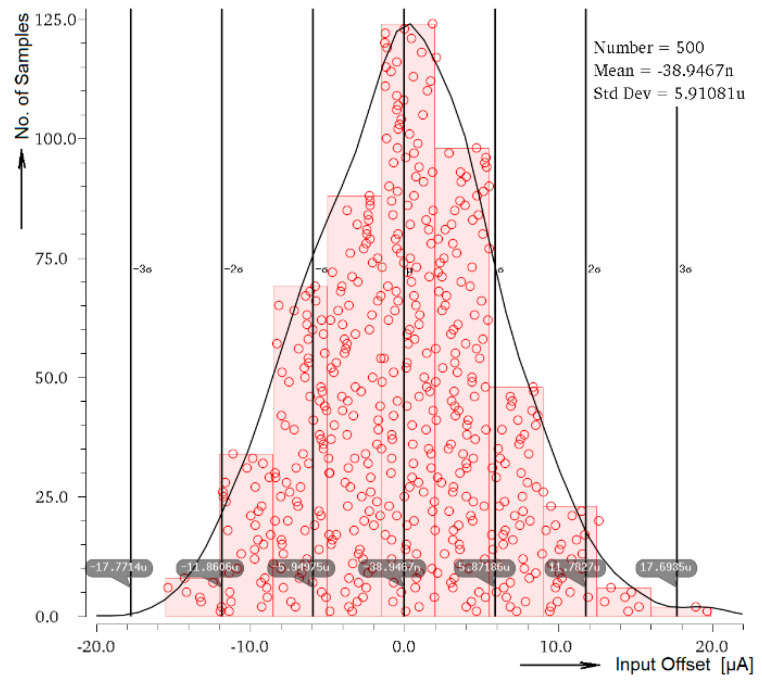
Monte Carlo input offset matching simulation.

Inspecting the results presented in Figure 6, there can be found out that systematic quiescent input offset (for *I*_IN_ = 0 A and B = 1) is equal to 5.4 nA, depending on the input signal amplitude it goes typically up to +0.8 µA at the edge of the input current operating range. As usual, even more important error is caused by the random offset, represented by the Monte Carlo matching analysis shown in Figure 14. In the histogram graph there the standard deviation 1σ = 5.9 µA is seen.

Another type of the offset analysis was executed at the level of simulation and measurement too. While input current is kept *I*_IN_ = 0 A, the *I*_CTRL_ was stepped in range (1 ÷ 20 µA) and the input current asymmetry observed. Whereas optimistic “systematic” offset simulation gives the offset from 5.4 nA to 60 nA (matching effect should be added), the measured offset acquired by the test is in range (4 ÷ 7) µA.

### 3.6. Input and Output Impedance

**Figure 15:**
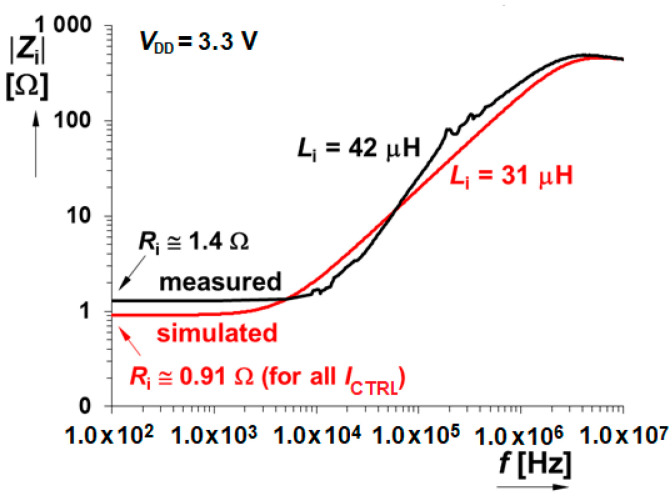
Input impedance frequency response.

Thanks to the used input stage topology the low input impedance represents an important advantage of this cell. It significantly helps to build current mode application circuits without additional errors or signal distortions. The compared simulation and measurement frequency responses of the input impedance are provided in Figure 15. The input impedance is very low (~1 Ω) at low frequencies. It started to increase above 10 kHz with still nice 18 Ω at 100 kHz. The also mentioned value of the serial inductance Li (the model of the input impedance includes the resistive and inductive parts in series with parallel connection of the input capacitance) gives us the information about the complex frequency response of the input impedance. The cut-off frequency $f_{0}=\frac{R_{i}}{2\pi L_{i}}$ defines the frequency where the input impedance starts to increase significantly with inductive character. For the referred values of the R_i_, L_i_ it can be calculated about 5 kHz as it is proved in Figure 15. The parallel capacitance causes the input impedance decrease at high frequencies but above the bandwidth of the presented circuit.

Also, the output impedance was determined with acceptable simulation to measurement difference. The simulated *R*_OUT_ = 8.8 MΩ for *I*_OUT_ = 0 A (4 MΩ in measurement) with 3dB decline at about 5 kHz. The DC output impedance is *R*_OUT_ = 7.5 MΩ when 100 µA is sourced/sinked from the output.

### 3.7. Result Overview

**Figure 16:**
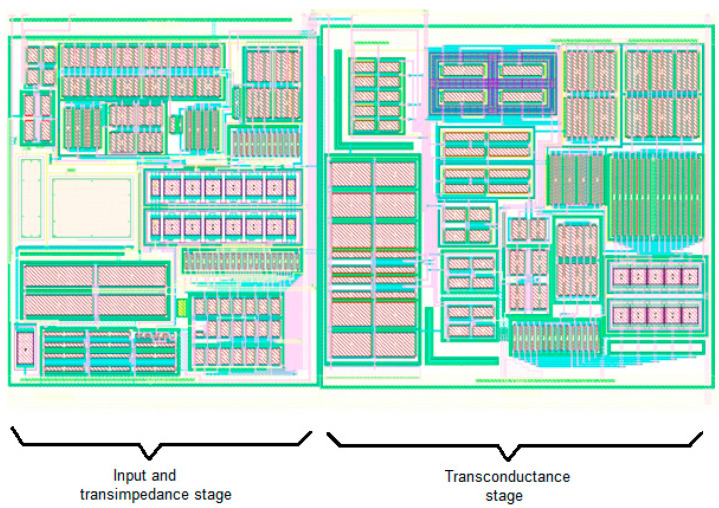
Layout of the presented amplifier.

The number of simulated and measured results confirms functionality of the designed circuit with assumed parameters. The tolerable differences between simulation and measurement are in the scope of the factory corner, given possibly by the uncertainty of the MOS transistor K_P_ transconductance parameter or, due to the nature of deviation, probably by the lower internal bias currents. Summary of the experimented circuit properties are published in Table 3. The illustrative Cadence layout is presented in Figure 16.

## 4. Discussion

The careful circuit analysis confirms that the newly developed modular topology of the designed tunable amplifier, working internally on the not very common fully linear principle, can be used with very interesting results. The good accordance between simulation results and measurement speaks about robustness of the introduced topology and of the whole design, as well. Acceptable deviations of measurements from simulations (decreasing gain and input range slightly but always in technology defined corners) are most likely caused by smaller internal bias against the ideal one. Moreover, the experimental measurement is influenced by increasing terminal and nodal parasitic capacities of ESD structures and PCB (approximately 10 pF), especially concerning the frequency response.

By observing the comparison Table 1, we can state that the presented circuit is almost the best one between the referred adjustable amplifiers in the maximum THD parameter. Only [14] reports lower harmonic distortion together with low power consumption, but with the digitally controlled topology, undefined input current range and even with purely theoretical results. Although the THD parameter is usually not the most important one for the amplifiers, it is reported only in limited number of publications. It can be seen that the discussed circuit is very good in the input/output impedance values and is at least well-comparable with other CMOS amplifiers regarding the input current range. In contrast, the BJT solutions are invincible in the input range and frequency bandwidth. However, their input/output impedances make them often unusable in the current mode signal processing chain for the precise measurement and their power consumption is not specified in the referred papers.

As could be expected in Table 2 comparing the current multipliers, much more references can be found stating the value of harmonic distortion. From this group only two circuits report slightly lower THD parameter than the presented one. The CMOS circuit from [65] also boasts the slightly better bandwidth and power consumption but its input current range is significantly lower. Furthermore, the terminal impedances are not reported. As the second one, the bipolar solution from [39] simultaneously shows significantly higher input range and bandwidth. This bipolar circuit was not realized and its impedances are not reported in the paper.

Even though the analyzed input offset is not really stunning, the presented circuit could be one of the better ones between current mode circuits with respect to precision. Unfortunately, there is no relevant comparison because most publications do not address this parameter. Furthermore, using an ADC in the modern sensor applications allows to easily subtract the DC offset from the signal. In that case the precise linearity is probably the most important parameter.

Despite the fact that this presented prototype was optimized especially for good linearity and accuracy properties, with the modified design it can be conveniently used in a wide range of applications from filters, precise generators to any signal processing where the signal multiplication is demanded. Specially in comparison with usual quadratic or exponential multiplier topologies it is able to report very good ratio of linear input range and power consumption.

The authors expect that the presented circuit is going to be beneficial in the field of sensor measurement and it could bring a little bit different approach to design of the precise adjustable circuits.

## Figures and Tables

**Table 1:** Comparison of features of adjustable current amplifiers or active devices including transfer response of adjustable current amplifier reported in recent works.

Reference Year of Publication	Adjustment (Analog, Dig., Res., Feedback)	Bandwidth [MHz]	Tested Current Gain Range [–]	Input Linearity Range [µA]	The Highest DC Input Resistance [Ω]	The Lowest DC Output Resistance [MΩ]	Power Consumption [mW]	THD [%]	Technology	Fabricated and Tested Experimentally
[3] 1988	A	30	0→30	N/A	N/A	N/A	N/A	N/A	BJT	Yes
[4] 1994	A	175	0.1→10	N/A	N/A	N/A	N/A	<1.9	BJT	Yes
[5] 2002	D	<100	0.12→1	N/A	N/A	N/A	N/A	N/A	CMOS AMI (1.2 µm)	No
[6] 2006	A	<100	1→3	±50	46	73	6.6	<2.5	CMOS TSMC (0.35 µm)	No
[7] 2008	A	<100	0.5→1	N/A	27000	0.175	N/A	N/A	BJT	No
[8] 2009	A	<10	0→20	N/A	<50	<31	N/A	N/A	CMOS (0.35 µm)	No
[9] 2010	A	<100	0.25→1	±5000	11	0.055	N/A	<0.6	BJT	Yes
[10] 2010	A	N/A	N/A	N/A	N/A	N/A	3.5	N/A	BJT	No
[11] 2012	A	<200	0.1→10	N/A	70	0.014	5.9	N/A	BJT	No
[12] * 2012	A	<100	0.1→8	±5000	adjust	0.055	N/A	<5	BJT	Yes
[13] * 2012	A	<40	0.5→3.5	±300	adjust	0.300	N/A	N/A	BJT + CMOS (ON 0.5 µm)	No
[14] 2013	D	<100	0.02→64	N/A	N/A	N/A	0.5	<0.15	CMOS (0.18 µm)	No
[15]* 2013 [17] 2016	D	<300	0.8→8	±300	5	0.1	10	N/A	CMOS ON (0.35 µm)	Yes
[18]* 2014	A	<25	0.36→3.61	±200	700	0.044	8.5	N/A	CMOS TSMC (0.18 µm)	No
[19]* 2017	A	<20	0→3.5	±1000	adjust	0.5	N/A	N/A	BJT	Yes
[20]* 2018	A	<70	0→1	±1400	adjust	0.055	N/A	N/A	BJT	No
[21,22] 2014	R	<1	1→16	N/A	50	10	0.28	<1	0.5 µm	Yes
[23] 2017	A	<68	1→23	N/A	624	0.060	1.73	5.9	CMOS TSMC (0.18 µm)	No
[24] 2019	R	<10	2.4→9.4	N/A	105	0.301	0.88	N/A	CMOS TSMC (0.25 µm)	No
[25] 2017	A	<300	0.1→6.3	±15	N/A	N/A	< 3	<1.8	CMOS (0.35 µm)	No
[26], 2006 [27] 2015	F	<500	N/A	±60	1	N/A	N/A	N/A	CMOS (0.8 µm)	No Yes
Prop. 2020	A	<0.5	0.00→1.7	±200	1.4	3.3	3.6	<0.35	CMOS ON (0.35 µm)	Yes

*—Prior work of some of the authors.

**Table 2:** Comparison of recent current-mode multipliers.

Reference Year of Publication	No. of Quadrants	Approximately Declared (Shown) Processed Input Levels [µA]	Ready for Immediate Practical Implementation without Additional Parts	Bandwidth [MHz]	Maximal THD [%]	Input Linearity Error [%]	The Highest DC Input Resistance [Ω]	The Lowest DC Output Resistance [MΩ]	Power Consumption [mW]	Technology (or Model)	Fabricated and Tested Experimentally
[28] 2000	4	±20	No	23	1.5	1.2	7000	N/A	0.93	CMOS 4007	Yes
[29] 1991	4	0–100	Yes	N/A	N/A	N/A	N/A	N/A	N/A	SPICE3C1	No
[30] 2009	4	±10	No	45	1.8	1.2	N/A	N/A	0.24	0.35 µm std. CMOS	No
[31] 2009	4	±200	No	3	N/A	N/A	N/A	N/A	N/A	SPICE BJT 2N2222+ +2N2907	No
[32] 2008	4	±10	No	N/A	N/A	N/A	N/A	N/A	N/A	SPICE BJT	No
[33] 2008	2/4	±60	Yes	114	5	N/A	N/A	N/A	3.8	AD844 + BJT	Yes
[34] 2007	4	±150	Yes	26	5.6	N/A	N/A	N/A	1.4	BJT AT&T	No
[35] 2006	4	±200	No	154	4	0.8	N/A	N/A	N/A	0.25 µm CMOS	No
[36] 2005	4	±0.25	No	0.2	0.9	5	N/A	N/A	0.006	0.35 µm CMOS	Yes
[37] 2004	4	±60	No	31	4.5	N/A	N/A	N/A	0.72	0.25 µm CMOS	No
[38] 2004	4	±25	No	N/A	0.5	0.4	N/A	N/A	N/A	2 µm MIETEC CMOS	No
[39] 2003	4	±1000	Yes	160	0.25	N/A	N/A	N/A	N/A	BJT 2N3904 2N3906	No
[40] 2002	4	±200	No	11	2	N/A	416	N/A	N/A	2 µm MOSIS SCNA	No
[41] 2001	4	±30	No	N/A	N/A	5	N/A	N/A	N/A	0.5 µm CMOS	Yes
[42] 2001	4	±50	Yes	33	N/A	0.9	N/A	N/A	0.6	0.5 µm CMOS	No
[43] 1999	2/4	0–200	No	16	0.9	N/A	N/A	N/A	N/A	0.7 µm MIETEC CMOS	No
[44] 2019	4	±0.02	No	0.16	3	N/A	N/A	N/A	N/A	0.35 µm AMS CMOS	No
[45] 2019	4	±0.5	No	0.1	N/A	N/A	N/A	N/A	0.018	0.18 µm standard CMOS	No
[46] 2019	4	±0.2	No	3.5	6	N/A	N/A	N/A	0.0005	0.065 µm std. CMOS	No
[47] 2018	4	±100	Yes	31	N/A	N/A	N/A	N/A	N/A	0.5 µm CMOS	No
[48] 2018	4	±10	Yes	460	1.2	N/A	N/A	N/A	0.8	0.18 µm CMOS	No
[49] 2017	4	±20	No	33	2.0	N/A	N/A	N/A	0.6	0.18 µm CMOS	No
[50] 2016	4	±10 0–200	No	75, 493	N/A	N/A	N/A	N/A	0.15	0.18 + 0.8 µm CMOS	No
[51] 2016	4	±10	No	493	N/A	N/A	N/A	N/A	0.15	0.18 µm CMOS	No
[52] 2016	4	±20	No	840	6	N/A	7200	N/A	0.09	0.18 µm std. CMOS	No
[53] 2015	4	±10	No	1320	1.1	1.0	N/A	N/A	0.09	0.25 µm CMOS	No
[54] 2015	1	0–10	No	N/A	N/A	N/A	N/A	N/A	0.32	0.18 µm CMOS	No
[55] 2015	4	±10	No	N/A	N/A	N/A	N/A	N/A	N/A	0.35 µm standard CMOS	No
[56] 2015	1	0–100	No	N/A	N/A	2	N/A	N/A	N/A	0.35 µm AMS CMOS	
[57] 2014	4	±8	No	180	1.3	1.5	7600	N/A	0.025	0.18 µm TSMC CMOS	No
[58] 2014	1	0–20	No	N/A	N/A	0.013	N/A	N/A	N/A	0.18 µm CMOS	No
[59] 2014	1	0–10	No	80	N/A	0.9	N/A	N/A	0.075	0.18 µm CMOS	No
[60] 2013	4	±20	No	N/A	2.5	3.5	N/A	N/A	6.4	0.5 µm CMOS	No
[61] 2013	2	±25	No	N/A	N/A	0.3	N/A	N/A	6.3	0.5 µm MIETEC CMOS	No
[62] 2012	4	unreadable	No	31	2.6	N/A	N/A	N/A	0.21	0.18 µm CMOS	No
[63] 2012	4	±30	No	3	1.1	0.3	N/A	N/A	0.0023	0.35 µm TSMC CMOS	No
[64] 2011	4	±100	Yes	N/A	4	N/A	N/A	N/A	N/A	BJT AT&T	No
[65] 2011	2	±25	No	3	0.2	N/A	N/A	N/A	2.0	0.5 µm CMOS	Yes
[66] 2010	2	±8	No	18	N/A	N/A	N/A	N/A	0.13	0.5 µm CMOS	Yes
[67] 2010	4	±200	Yes	N/A	N/A	N/A	N/A	N/A	N/A	BJT AT&T	No
[68] 2009	4	±150	Yes	53	4.3	N/A	N/A	N/A	1.8	BJT AT&T	No
Prop 2020	2	±200	Yes	0.5	0.35	5.4	1.4	3	3.6	ON I3T25/035 CMOS	Yes

**Table 3:** Amplifier properties summary.

Parameter	Simulated (Nominal)	Measured (Selected Prototype)
small-signal gain and AC transfer bandwidth (−3 dB)		
B(i) for *I*_CTRL_ = 1 µA	0.076 [–], −22.3 dB (0.69 MHz)	0.067 [–], −23.4 dB (0.46 MHz)
B(i) for *I*_CTRL_ = 2.5 µA	0.189 [–], −14.5 dB (0.95 MHz)	0.169 [–], −15.5 dB (0.64 MHz)
B(i) for *I*_CTRL_ = 7.5 µA	0.566 [–], −4.9 dB (1.24 MHz)	0.519 [–], −5.7 dB (0.83 MHz)
B(i) for *I*_CTRL_ = 12.5 µA	0.994 [–], −0.05 dB (1.51 MHz)	0.912 [–], −0.8 dB (0.90 MHz)
B(i) for *I*_CTRL_ = 17.5 µA	1.632 [–], 4.3 dB (2.89 MHz)	1.122 [–], 1.0 dB (0.94 MHz)
B(i) for *I*_CTRL_ = 20.0 µA	2.405 [–], 7.6 dB (3.00 MHz)	1.73 [–], 4.76 dB (1.00 MHz)
BW for *I*_CTRL_ = 1→20 µA	0.69→3.00 MHz	0.46→1.0 MHz
input DC dynamic range		
for ICTRL= 1→20 µA	−200→200 µA	−190→180 µA
input signal distortion (full input signal range)		
Linearity (*I*_CTRL_ = 1→20 µA)	1.8%	6%
THD (*I*_CTRL_ = 1→20 µA)	0.227%	0.35%
gain control input linearity (*I*_IN_ = 100 µA)		
Linearity (*I*_CTRL_ = 1→12.5 µA)	10%	N/A
THD (*I*_CTRL_ = 1→12.5 µA)	3%	N/A
input DC offset (*I*_IN_→0 A)		
systematic (*I*_CTRL_ = 1→20 µA)	5.4 nA→60 nA	N/A
MC matching offset	σ = 5.9 µA	N/A
measured	N/A	4→−7 µA
input/output impedances		
Ri, (Li)	0.91 Ω (31 µH)	1.4 Ω (42 µH)
Ro, (Co)	8.8 MΩ (3.9 pF)	4 MΩ (14.1 pF]
consumption		
*I*_VDD_ (*I*_OUT_ = −200, 0, +200 µA)	1.35 mA, 1.1 mA, 0.87 mA	N/A *
Pd (for *V*_DD_ = 3.3 V)	4.45 mW, 3.63 mW, 2.88 mW	N/A *

* Could not be measured as the amplifier is a part of the prototype chip with common supply.
